# Antibacterial Utilization for Febrile Illnesses and Laboratory-Confirmed Bloodstream Infections in Northern Tanzania

**DOI:** 10.1093/ofid/ofad448

**Published:** 2023-08-21

**Authors:** Ganga S Moorthy, Deng B Madut, Kajiru G Kilonzo, Bingileki F Lwezaula, Ronald Mbwasi, Blandina T Mmbaga, James S Ngocho, Wilbrod Saganda, John P Bonnewell, Manuela Carugati, Joseph R Egger, Julian T Hertz, L Gayani Tillekeratne, Michael J Maze, Venance P Maro, John A Crump, Matthew P Rubach

**Affiliations:** Division of Pediatric Infectious Diseases, Department of Pediatrics, Duke University Medical Center, Durham, North Carolina, USA; Duke Global Health Institute, Duke University, Durham, North Carolina, USA; Duke Global Health Institute, Duke University, Durham, North Carolina, USA; Division of Infectious Diseases and International Health, Department of Medicine, Duke University Medical Center, Durham, North Carolina, USA; Kilimanjaro Christian Medical Centre-Duke University Collaboration, Kilimanjaro Christian Medical Centre, Moshi, Tanzania; Kilimanjaro Christian Medical University College, Tumaini University, Moshi, Tanzania; Department of Medicine, Mawenzi Regional Referral Hospital, Moshi, Tanzania; Kilimanjaro Christian Medical Centre-Duke University Collaboration, Kilimanjaro Christian Medical Centre, Moshi, Tanzania; Kilimanjaro Christian Medical Centre-Duke University Collaboration, Kilimanjaro Christian Medical Centre, Moshi, Tanzania; Kilimanjaro Christian Medical University College, Tumaini University, Moshi, Tanzania; Kilimanjaro Christian Medical Centre-Duke University Collaboration, Kilimanjaro Christian Medical Centre, Moshi, Tanzania; Kilimanjaro Christian Medical University College, Tumaini University, Moshi, Tanzania; Kilimanjaro Christian Medical University College, Tumaini University, Moshi, Tanzania; Duke Global Health Institute, Duke University, Durham, North Carolina, USA; Division of Infectious Diseases and International Health, Department of Medicine, Duke University Medical Center, Durham, North Carolina, USA; Division of Infectious Diseases and International Health, Department of Medicine, Duke University Medical Center, Durham, North Carolina, USA; Duke Global Health Institute, Duke University, Durham, North Carolina, USA; Duke Global Health Institute, Duke University, Durham, North Carolina, USA; Duke Global Health Institute, Duke University, Durham, North Carolina, USA; Division of Infectious Diseases and International Health, Department of Medicine, Duke University Medical Center, Durham, North Carolina, USA; Centre for International Health, University of Otago, Dunedin, New Zealand; Department of Medicine, University of Otago, Christchurch, New Zealand; Kilimanjaro Christian Medical Centre-Duke University Collaboration, Kilimanjaro Christian Medical Centre, Moshi, Tanzania; Kilimanjaro Christian Medical University College, Tumaini University, Moshi, Tanzania; Duke Global Health Institute, Duke University, Durham, North Carolina, USA; Division of Infectious Diseases and International Health, Department of Medicine, Duke University Medical Center, Durham, North Carolina, USA; Kilimanjaro Christian Medical University College, Tumaini University, Moshi, Tanzania; Centre for International Health, University of Otago, Dunedin, New Zealand; Duke Global Health Institute, Duke University, Durham, North Carolina, USA; Division of Infectious Diseases and International Health, Department of Medicine, Duke University Medical Center, Durham, North Carolina, USA

**Keywords:** antibacterial use, antimicrobial stewardship, fever, guidelines, Tanzania

## Abstract

**Background:**

We describe antibacterial use in light of microbiology data and treatment guidelines for common febrile syndromes in Moshi, Tanzania.

**Methods:**

We compared data from 2 hospital-based prospective cohort studies, cohort 1 (2011–2014) and cohort 2 (2016–2019), that enrolled febrile children and adults. A study team member administered a standardized questionnaire, performed a physical examination, and collected blood cultures. Participants with bloodstream infection (BSI) were categorized as receiving effective or ineffective therapy based upon antimicrobial susceptibility interpretations. Antibacterials prescribed for treatment of pneumonia, urinary tract infection (UTI), or presumed sepsis were compared with World Health Organization and Tanzania Standard Treatment Guidelines. We used descriptive statistics and logistic regression to describe antibacterial use.

**Results:**

Among participants, 430 of 1043 (41.2%) and 501 of 1132 (44.3%) reported antibacterial use prior to admission in cohorts 1 and 2, respectively. During admission, 930 of 1043 (89.2%) received antibacterials in cohort 1 and 1060 of 1132 (93.6%) in cohort 2. Inpatient use of ceftriaxone, metronidazole, and ampicillin increased between cohorts (*P* ≤ .002 for each). BSI was detected in 38 (3.6%) participants in cohort 1 and 47 (4.2%) in cohort 2. Of 85 participants with BSI, 81 (95.3%) had complete data and 52 (64.2%) were prescribed effective antibacterials. Guideline-consistent therapy in cohort 1 and cohort 2 was as follows: pneumonia, 87.4% and 56.8%; UTI, 87.6% and 69.0%; sepsis, 84.4% and 61.2% (*P* ≤ .001 for each).

**Conclusions:**

Receipt of antibacterials for febrile illness was common. While guideline-consistent prescribing increased over time, more than one-third of participants with BSI received ineffective antibacterials.

## Teaser

Receipt of antibacterials prior to and during hospital admission was common within 2 large cohorts of febrile inpatients in northern Tanzania. While guideline-consistent prescribing improved over time, a large proportion of participants with bloodstream infection received ineffective antibacterials.

Fever is a common reason for seeking healthcare in low- and middle-income countries (LMICs). In such settings, clinical diagnosis is difficult and access to diagnostic methods can be limited. The diagnosis and treatment of specific causes of febrile illness often occur based on clinical suspicion, and broad-spectrum antibacterials are commonly prescribed empirically [1–3]. Empiric antimicrobial use is often appropriate when there is high suspicion for bacterial infection or incipient severe illness. However, widespread use of broad-spectrum antibacterials is common in LMICs and contributes to the major public health concern of antimicrobial resistance [4–7].

Antibacterial prescribing practices and the positive impact of antimicrobial stewardship programs are well documented in high-income countries [8, 9]. LMICs face unique challenges that hinder the implementation of antimicrobial stewardship programs, including overuse of antimicrobials in the community and in hospitals, limited access to therapeutics in some areas, underuse and distrust of laboratory services, and limited laboratory and human resources [10, 11]. However, despite these barriers, several countries in sub-Saharan Africa have successfully implemented both community- and hospital-based antimicrobial stewardship programs [12]. In Tanzania, although national guidelines exist, adherence to standard treatment guidelines (STGs) varies among regions, with studies reporting levels of adherence ranging from 15% to 84% [13–15]. To advance antimicrobial stewardship programs in Tanzania, further data are needed on empiric antibacterial prescribing patterns both prior to and during hospital care, use of appropriate and targeted therapy for laboratory-confirmed infections, and adherence to available treatment guidelines [16].

To provide insight into antimicrobial prescribing practices in northern Tanzania, we conducted an analysis of patients enrolled in prospective hospital-based fever surveillance studies in Moshi, Tanzania. We describe use of antibacterial therapy in this patient population, use of effective therapy for laboratory-confirmed bloodstream infections (BSIs), and adherence to published guidelines for common febrile syndromes.

## MATERIALS AND METHODS

### Study Aims

The primary aim of this study was to describe antibacterial prescribing patterns in participants with febrile illnesses in northern Tanzania. Secondary aims were (1) in participants with BSIs, to assess concordance of antibacterial therapy with the organism's drug susceptibility, and (2) in participants with nonstudy clinician–furnished diagnoses of pneumonia, urinary tract infection (UTI), or sepsis, to describe concordance of prescribed antibacterials with published pediatric and adult STGs.

### Setting

Our analysis utilized data from hospital-based fever surveillance studies conducted at Kilimanjaro Christian Medical Centre (KCMC) and Mawenzi Regional Referral Hospital (MRRH) in Moshi, Tanzania. The prospective studies occurred from 26 September 2011 through 31 May 2014 (cohort 1) and 7 September 2016 through 31 May 2019 (cohort 2). Results from these studies have been previously described [17–19]. Moshi has a population of >180 000 and is at an elevation of approximately 890 meters above sea level [20]. KCMC is a zonal referral hospital with 630 inpatient beds serving the regions of Kilimanjaro, Tanga, Arusha, Manyara, Dodoma, and Singida in northern Tanzania. MRRH is a regional hospital with 300 inpatient beds serving the Kilimanjaro region.

### Study Participants

Adults aged ≥13 years admitted to both KCMC and MRRH and children aged 2–12 years and infants aged 0–1 years admitted to MRRH were enrolled in cohort 1. Participants were also enrolled in outpatient clinics in cohort 1, but we excluded them from our analyses. Admitted adults, children, and infants at both hospitals were enrolled in cohort 2. Inpatients were eligible for enrollment if they had a documented fever defined as tympanic temperature of ≥38.0°C or a history of fever within the previous 72 hours. All patients were screened and enrolled within 24 hours after admission.

### Study Procedures

After obtaining informed consent, a trained study team member administered a standardized questionnaire recording demographics, history of illness, and antimicrobial use. They also performed a physical examination. The study team attempted blood culture collection on all participants within 24 hours of admission. We recorded participant self-reported human immunodeficiency virus (HIV) status in the 2011–2014 period, and during the 2016–2019 study period offered testing to participants using the SD Bioline (Abbott Laboratories, Abbott Park, Illinois) HIV-1/2 3.0 test with confirmation by a second rapid test, Uni-Gold (Trinity Biotech, Bray, Ireland). A study team member recorded antimicrobials prescribed during the hospitalization, participant vital status at discharge, and preliminary and discharge diagnoses furnished by the treating, nonstudy clinician(s) using the *International Classification of Diseases, Tenth Revision*.

### Laboratory Methods

Full details of blood culture collection procedures have been previously published [21]. In brief, BacT/ALERT Pediatric FAN or BacT/ALERT Pediatric FAN Plus and BacT/ALERT Standard Aerobic or BacT/ALERT Standard Aerobic Plus (bioMérieux, Marcy l’Etoile, France) bottles were used for pediatric and adult patients, respectively. We monitored blood culture fill volumes to ensure adequate sample collection. Blood cultures were analyzed using the BacTAlert 3D Microbial Detection system (bioMérieux) with a maximum incubation period of 5 days. Standard methods were used for identifying bloodstream isolates [22]. Antimicrobial susceptibility testing was manually performed according to the methods of the Clinical and Laboratory Standards Institute (CLSI, Wayne, Pennsylvania). Susceptibility interpretations were based on the 2019 CLSI guidelines and interpretive criteria, classified as susceptible, intermediate, or resistant [23].

### Definitions

Prior antibacterial and prior antimalarial use were defined as self-reported usage of medications for the present illness prior to admission to either KCMC or MRRH. The use of trimethoprim-sulfamethoxazole prophylaxis for prevention of HIV-associated opportunistic infections was also self-reported by participants and such use was not included in the tally of prior antibacterial use for treatment of the present illness. Inpatient antibacterial use was defined as antibacterials prescribed after admission. Among the antibacterials recorded on study case report forms (see Supplementary Table 1), the following were categorized as broad spectrum based on published frameworks: ceftriaxone, ciprofloxacin, amoxicillin-clavulanate, or azithromycin [17].

Among participants with BSIs, 2 authors with clinical training in infectious diseases (G. S. M. and M. P. R.) determined effectiveness of prescribed antibacterials through manual data review and discussion to ensure agreement. Effective therapy was defined as receiving an antibacterial with demonstrated activity based upon phenotypic antimicrobial susceptibility testing of the isolate or based upon CLSI guidance that susceptibility can be assumed. Ineffective therapy was defined as 1 or more of the following: (1) participant was prescribed an antibacterial without adequate activity against the microbiologically identified organism or intrinsic resistance; (2) no antimicrobial was prescribed to the participant; (3) enteral therapy was prescribed for a clinical scenario where enteral therapy was not indicated; and/or (4) the microbiologically identified organism generally has susceptibility to the prescribed antibacterial but the specific isolate demonstrated resistance against the antibacterial the participant was prescribed.

Antimicrobials prescribed for preliminary or final diagnosis of pneumonia, UTI, or presumed sepsis were compared with syndrome- and age-specific recommendations from the World Health Organization (WHO) and the Tanzania STGs and National Essential Medicines List, fourth edition (2013) and fifth edition (2017) (Supplementary Table 2) [24–28]. In participants with the aforementioned clinical conditions, we assessed for guideline-consistent therapy, defined as inpatient antibacterial selection consistent with recommendations of either the WHO or Tanzania STGs. We then assessed country-specific guideline consistency with the recommendations of the Tanzania STGs.

### Statistical Analysis

Statistical analyses were performed with R software, version 4.1.1. Descriptive statistics were performed for demographics and baseline participant characteristics. Continuous variables were expressed using median and interquartile range or mean and standard deviation. Categorical variables are expressed as frequencies. Differences between the 2 study periods were assessed using Pearson χ^2^ test for categorical variables and the Wilcoxon rank-sum test for continuous variables. Statistical significance was evaluated at *P* values <.05.

Multivariable logistic regression analyses were performed to identify factors associated with antibacterial prescription prior to enrollment and predictors of broad-spectrum inpatient antibacterial administration. Univariable logistic regression was performed to assess for a relationship between ineffective antimicrobial therapy for BSI and in-hospital mortality.

To understand temporal trends in guideline-consistent treatment of pneumonia, UTI, and sepsis, predictive margins with 95% confidence intervals (CIs) were computed. First, we constructed logistic regression models with the dependent variables as guideline-consistent therapy for each syndrome and the explanatory variable as time in years adjusted for participant age in years, sex, and hospital location. Time zero was assigned to the first date of enrollment in cohort 1. We then plotted the predicted probabilities of guideline-consistent therapy in 1-year increments.

### Research Ethics

The febrile surveillance studies were approved by the Kilimanjaro Christian Medical University College of Tumaini University Health Research Ethics Committee, the Tanzania National Institute for Medical Research National Health Research Ethics Coordinating Committee, and an Institutional Review Board of the Duke University Health System. Written informed consent was obtained from all participants or from the participant's parent or guardian.

## RESULTS

### Sociodemographic and Clinical Characteristics of Study Participants

Demographic and clinical characteristics of participants with febrile illness at KCMC and MRRH are shown in Table 1. A total of 34 184 inpatients were screened (15 305 in cohort 1 and 18 879 in cohort 2). Among those screened, 5461 were eligible for enrollment: 1837 in cohort 1 and 3624 in cohort 2. A total of 2182 participants were enrolled: 1050 in cohort 1 and 1132 in cohort 2. We analyzed data from 2175 enrolled participants: 1043 in cohort 1 and 1132 in cohort 2. There were 30 (2.9%) in-hospital deaths in cohort 1 and 66 (5.8%) in cohort 2.

**Table 1.** Demographic, Clinical, and Laboratory Characteristics of Participants With Febrile Illness Enrolled in 2 Fever Surveillance Studies, Northern Tanzania, 2011–2014 and 2016–2019

Characteristic	Cohort 1 (2011–2014)	Cohort 2 (2016–2019)	*P* Value	Total
No. of participants enrolled	1043	1132		2175
Age, y, median (IQR)	29 (5–41)	22 (2–45)	.122	26 (3–43)
Adults (≥13 y)	729 (69.9)	653 (57.7)	<.001	1382 (63.5)
Children (2–12 y)	241 (23.1)	320 (28.3)		561 (25.8)
Infants (0–1 y)	73 (7.0)	159 (14.0)		232 (10.7)
Female	569 (54.6)	518 (45.8)	<.001	1087 (50.0)
Death during admission	30 (2.9)	66 (5.8)	.001	96 (4.4)
Days of illness, median (IQR)	7 (3–14)	5 (3–11)	<.001	5 (3–14)
Days of fever, median (IQR)	4 (3–7)	3 (2–7)	<.001	4 (2–7)
HIV infection confirmed by testing^a^	Not done	196 (17.3)	…	…
Trimethoprim-sulfamethoxazole prophylaxis	117 (11.2)	114 (10.1)	.386	231 (10.6)
Reported use of antimalarials prior to admission	353 (33.8)	122 (10.8)	<.001	475 (22.0)
Reported use of antibacterials prior to admission	430 (41.2)	501 (44.3)	.161	931 (42.8)
Positive malaria parasite smear	23 (2.2)	42 (3.7)	.046	65 (3.0)
Laboratory-confirmed bacteremia	38 (3.6)	47 (4.2)	.546	85 (3.9)
Prescribed inpatient HIV antiretroviral medications	125 (12.0)	147 (13.0)	.544	272 (12.5)
Prescribed inpatient treatment for tuberculosis	41 (4.0)	25 (2.2)	.025	66 (3.0)
Prescribed inpatient antibacterials for febrile illness	930 (89.2)	1060 (93.6)	<.001	1990 (91.5)
Prescribed inpatient broad-spectrum antibacterials	548 (52.5)	682 (60.2)	<.001	1230 (56.6)

Data are presented as No. (%) unless otherwise indicated.

Abbreviations: HIV, human immunodeficiency virus; IQR, interquartile range.

a HIV status was obtained by self-report in cohort 1 and by HIV rapid antibody test in cohort 2.

### Antibacterial Treatments Reportedly Used by Participants Prior to Admission to KCMC or MRRH

Antibacterial use prior to admission to KCMC or MRRH is reported in Table 2. There were higher odds of antibacterial use for infants (odds ratio [OR], 1.65 [95% CI, 1.23–2.20]) and children (OR, 1.43 [95% CI, 1.16–1.75]) compared to adults. Participants in cohort 2 had increased odds of reporting antibacterial use (OR, 1.21 [95% CI, 1.02–1.45]) compared to cohort 1 participants.

**Table 2.** Antibacterials Used Prior to Admission to Kilimanjaro Christian Medical Centre or Mawenzi Regional Referral Hospital as Reported by Study Participants With Febrile Illness, Northern Tanzania, 2011–2014 and 2016–2019

Characteristic	Cohort 1 (2011–2014)			Cohort 2 (2016–2019)			Total		
	Adults	Children	Infants	Adults	Children	Infants	Adults	Children	Infants
No. of participants enrolled	729	241	73	653	320	159	1382	561	232
Participants reporting antibacterial use prior to admission, No. (%)	300 (41.2)	101 (41.9)	29 (39.7)	258 (39.5)	156 (48.8)	87 (54.7)	558 (40.4)	257 (45.8)	116 (50.0)
No. of prior antibacterials per person, mean (SD)	1.3 (0.7)	1.2 (0.4)	1.4 (0.6)	1.3 (0.9)	1.2 (0.9)	1.4 (0.9)	1.3 (0.8)	1.2 (0.7)	1.4 (0.9)
Type of antibacterial reported as used prior to admission, No. (%)									
Ampicillin	9 (1.2)	1 (0.4)	3 (4.1)	7 (1.1)	11 (3.4)	12 (7.5)	16 (1.2)	12 (2.1)	15 (6.5)
Ampicillin-cloxacillin	9 (1.2)	7 (2.9)	0 (0.0)	15 (2.3)	12 (3.8)	5 (3.1)	24 (1.7)	19 (3.4)	5 (2.2)
Amoxicillin^a^	…			47 (7.2)	33 (10.3)	18 (11.3)	…		
Amoxicillin-clavulanate	72 (9.9)	29 (12.0)	7 (9.6)	9 (1.4)	10 (3.1)	10 (6.3)	81 (5.9)	39 (7.0)	17 (7.3)
Cloxacillin	12 (1.6)	2 (0.8)	2 (2.7)	0 (0.0)	1 (0.3)	2 (1.3)	12 (0.9)	3 (0.5)	4 (1.7)
Penicillin	17 (2.3)	7 (2.9)	4 (5.5)	18 (2.8)	11 (3.4)	10 (6.3)	35 (2.5)	18 (3.2)	14 (6.0)
Cephalexin^a^	…			4 (0.6)	8 (2.5)	2 (1.3)	…		
Ceftriaxone	12 (1.6)	1 (0.4)	0 (0.0)	42 (6.4)	15 (4.7)	8 (5.0)	54 (3.9)	16 (2.9)	8 (3.4)
Trimethoprim-sulfamethoxazole^b^	25 (3.4)	24 (10.0)	6 (8.2)	9 (1.4)	16 (5.0)	9 (5.6)	34 (2.5)	40 (7.1)	15 (6.4)
Doxycycline	12 (1.6)	0 (0.0)	0 (0.0)	14 (2.1)	3 (0.9)	0 (0.0)	26 (1.9)	3 (0.5)	0 (0.0)
Chloramphenicol	7 (1.0)	1 (0.4)	1 (1.4)	0 (0.0)	1 (0.3)	1 (0.6)	7 (0.5)	2 (0.4)	2 (0.9)
Azithromycin	8 (1.1)	2 (0.8)	1 (1.4)	16 (2.5)	10 (3.1)	7 (4.4)	24 (1.7)	12 (2.1)	8 (3.4)
Erythromycin	10 (1.4)	13 (5.4)	7 (9.6)	4 (0.6)	14 (4.4)	9 (5.7)	14 (1.0)	27 (4.8)	16 (6.9)
Gentamicin	6 (0.8)	2 (0.8)	1 (1.4)	10 (1.5)	13 (4.1)	13 (8.2)	16 (1.2)	15 (2.7)	14 (6.0)
Ciprofloxacin	16 (2.2)	0 (0.0)	0 (0.0)	32 (4.9)	1 (0.3)	0 (0.0)	48 (3.5)	1 (0.2)	0 (0.0)
Metronidazole	34 (4.7)	6 (2.5)	3 (4.1)	46 (7.0)	16 (5.0)	5 (3.1)	80 (5.8)	22 (3.9)	8 (3.4)
Other antibacterial	22 (3.0)	8 (3.3)	7 (9.6)	4 (0.6)	5 (1.6)	3 (1.9)	26 (1.9)	13 (2.3)	10 (4.3)
Unknown antibacterial	11 (1.5)	3 (1.2)	1 (1.4)	11 (1.7)	2 (0.6)	0 (0.0)	22 (1.6)	5 (0.9)	1 (0.4)

Abbreviations: SD, standard deviation.

a Case report form in cohort 1 did not collect data regarding use of amoxicillin and cephalexin prior to admission.

b Used for therapy rather than disease prophylaxis among persons with human immunodeficiency virus.

### Antibacterials Prescribed to Participants During Admission to KCMC or MRRH

In cohort 1, 930 (89.2%) participants received antibacterials during admission for febrile illness compared with 1060 (93.6%) in cohort 2 (*P* < .001) (Table 3). Among participants receiving inpatient antibacterials, 548 (52.5%) in cohort 1 and 682 (60.2%) in cohort 2 (*P* ≤ .001) received broad-spectrum therapy. Use of ceftriaxone, metronidazole, and ampicillin increased from cohort 1 to cohort 2 (ceftriaxone *P* ≤ .001; metronidazole *P* = .020; ampicillin *P* = < .001) (Table 3).

**Table 3.** Characteristics of Antibacterials Prescribed to Study Participants During Admission at Kilimanjaro Christian Medical Centre or Mawenzi Regional Referral Hospital, Northern Tanzania, 2011–2014 and 2016–2019

Characteristic	Cohort 1 (2011–2014)			Cohort 2 (2016–2019)			Total		
	Adults	Children	Infants	Adults	Children	Infants	Adults	Children	Infants
No. of participants enrolled	729	241	73	653	320	159	1382	561	232
Participants prescribed antibacterials inpatient, No. (%)	621 (85.2)	237 (98.3)	72 (98.6)	597 (91.4)	309 (96.6)	154 (96.9)	1218 (88.1)	546 (97.3)	226 (7.4)
Number of inpatient antibacterials prescribed per participant, mean (SD)	2.2 (1.1)	2.4 (1.0)	2.5 (1.2)	2.6 (1.3)	3.3 (1.4)	3.4 (1.4)	2.4 (1.2)	2.9 (1.3)	3.1 (1.4)
Participants prescribed broad-spectrum antibacterials, No. (%)	409 (56.1)	113 (46.9)	26 (35.6)	476 (72.9)	146 (45.6)	60 (37.7)	885 (64.0)	259 (46.2)	86 (37.1)
Type of antibacterial prescribed inpatient, No. (%)									
Ampicillin	59 (8.1)	43 (17.8)	21 (28.8)	111 (17.0)	204 (63.7)	112 (70.4)	170 (12.3)	247 (44.0)	133 (57.3)
Ampicillin-cloxacillin	38 (5.2)	21 (8.7)	6 (8.2)	26 (4.0)	33 (10.3)	25 (15.7)	64 (4.6)	54 (9.6)	31 (13.4)
Amoxicillin^a^	…			1 (0.2)	3 (0.9)	5 (3.1)	…		
Amoxicillin-clavulanate	151 (20.7)	32 (13.3)	3 (4.1)	60 (9.2)	49 (15.3)	18 (11.3)	211 (15.3)	81 (14.4)	21 (9.1)
Cloxacillin	28 (3.8)	11 (4.6)	2 (2.7)	56 (8.6)	79 (24.7)	45 (28.3)	84 (6.1)	90 (16.0)	47 (20.3)
Penicillin	161 (22.1)	72 (29.9)	16 (21.9)	16 (2.5)	15 (4.7)	2 (1.3)	177 (12.8)	87 (15.5)	18 (7.8)
Cephalexin	9 (1.2)	1 (0.4)	1 (0.1)	8 (1.2)	10 (3.1)	3 (1.9)	17 (1.2)	11 (2.0)	4 (1.7)
Ceftriaxone	211 (28.9)	57 (23.7)	14 (19.2)	409 (62.6)	97 (30.3)	43 (27.0)	620 (44.9)	154 (27.5)	57 (24.6)
Trimethoprim-sulfamethoxazole^b^	110 (15.1)	76 (31.5)	31 (42.5)	71 (10.9)	14 (4.4)	4 (2.5)	181 (13)	90 (16)	35 (15.1)
Doxycycline	21 (2.9)	0 (0.0)	0 (0.0)	7 (1.1)	1 (0.3)	0 (0.0)	28 (2.0)	1 (0.2)	0 (0.0)
Chloramphenicol	32 (4.4)	42 (17.4)	13 (17.8)	3 (0.5)	15 (4.7)	5 (3.1)	35 (2.5)	57 (10.2)	18 (7.8)
Azithromycin	64 (8.8)	32 (13.3)	10 (13.7)	39 (6.0)	10 (3.1)	7 (4.4)	103 (7.5)	42 (7.5)	17 (7.3)
Erythromycin	32 (4.4)	14 (5.8)	5 (6.8)	14 (2.1)	3 (0.9)	3 (1.9)	46 (3.3)	17 (3.0)	8 (3.4)
Gentamicin	50 (6.9)	64 (26.6)	25 (34.2)	61 (9.3)	193 (60.3)	117 (73.6)	111 (8.0)	257 (45.8)	142 (61.2)
Ciprofloxacin	64 (8.8)	3 (1.2)	0 (0.0)	72 (11.0)	5 (1.6)	0 (0.0)	136 (9.8)	8 (1.4)	0 (0.0)
Metronidazole	148 (20.3)	43 (17.8)	17 (23.3)	183 (28.0)	80 (25.0)	10 (6.3)	331 (24.0)	123 (21.9)	27 (11.6)
Other antibacterial	77 (10.6)	31 (12.9)	16 (21.9)	24 (3.7)	12 (3.8)	2 (1.3)	101 (7.3)	43 (7.7)	18 (7.8)

Abbreviations: SD, standard deviation.

a Case report form in cohort 1 collected data regarding prescription of inpatient amoxicillin.

b Used for therapy rather than disease prophylaxis among persons with human immunodeficiency virus.

### Antibacterial Prescription Among Admitted Participants With BSI

BSI was identified in 38 (3.6%) cohort 1 participants and in 47 (4.2%) cohort 2 participants. Complete data to determine effectiveness of antibacterial prescription were available for 81 of the 85 (95.3%) participants (38 in cohort 1 and 43 in cohort 2). Overall, 52 of 81 (64.2%) participants were prescribed effective therapy (Supplementary Table 3). In cohort 1, 25 (65.8%) participants were prescribed effective therapy and 13 (34.2%) ineffective therapy. Among the 13 who were prescribed ineffective therapy, 3 (7.9%) participants were prescribed antibacterial therapy to which the isolate exhibited phenotypic resistance. In cohort 2, 27 (62.8%) participants were prescribed effective therapy and 16 (37.2%) ineffective therapy. Five of the 43 (11.6%) participants with BSI were prescribed antibacterial therapy to which the isolate exhibited phenotypic resistance. Further details regarding susceptibility for selected isolates are shown in Supplementary Table 4.

Among the 85 participants with BSI, 8 (9.4%) died while hospitalized and, of these, 4 (50.0%) received ineffective therapy. Odds of in-hospital mortality for ineffective therapy were 0.52 (95% CI, .11–2.37).

### Consistency With Global and National Standard Treatment Guidelines

**Figure 1.**
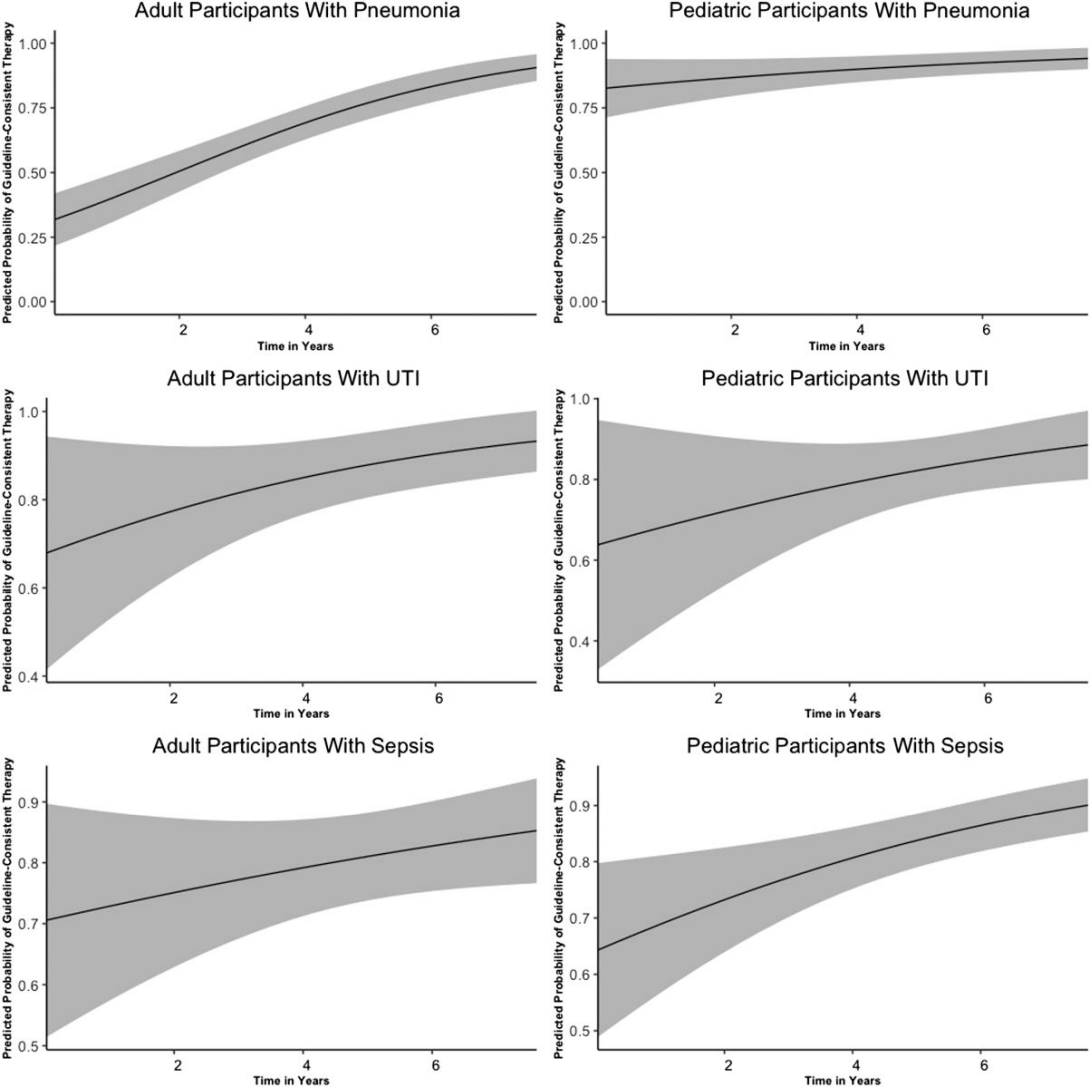
Adjusted prediction of guideline-consistent therapy among hospitalized patients with pneumonia, urinary tract infection (UTI), and sepsis in 2 febrile surveillance studies from September 2011 through May 2019. Time zero was assigned to the first date of enrollment in cohort 1. The black line displays the mean; gray areas represent the 95% confidence interval.

Consistency of prescribed therapy with WHO or Tanzanian STGs, and consistency with the Tanzanian STGs alone, are shown in Table 4. We observed an increase in WHO or Tanzanian STG-consistent therapy for pneumonia, UTI, and sepsis over time (Figure 1).

**Table 4.** Consistency of Inpatient Antibacterial Prescription With World Health Organization and/or Tanzanian Standard Treatment Guidelines Among Participants Enrolled in 2 Fever Surveillance Studies, Northern Tanzania, 2011–2014 and 2016–2019

Diagnosis and Guideline	Cohort 1 (2011–2014)		Cohort 2 (2016–2019)		Total, No.
	Pediatric (<13 y)	Adult (≥13 y)	Pediatric (<13 y)	Adult (≥13 y)	
Diagnosis of pneumonia, No.	83	130	195	139	547
Therapy consistent with either WHO or Tanzanian STGs	65 (78.3)	56 (43.0)	177 (90.8)	115 (82.7)	413
Therapy consistent with Tanzanian STGs	61 (73.5)	53 (40.8)	167 (85.6)	106 (76.3)	387
Diagnosis of UTI, No.	15	27	84	69	195
Therapy consistent with either WHO or Tanzanian STGs	12 (80.0)	18 (66.7)	73 (86.9)	63 (91.3)	166
Therapy consistent with Tanzanian STGs	2 (13.3)^a^	8 (29.6)	5 (6.0)^a^	22 (31.9)	37
Diagnosis of sepsis, No.	20	34	111	101	266
Therapy consistent with either WHO or Tanzanian STGs	14 (70.0)	22 (64.7)	95 (85.6)	84 (83.2)	215
Therapy consistent with Tanzanian STGs	14 (70.0)	22 (64.7)	95 (85.6)	84 (83.2)	215

Data are presented as No. (%) unless otherwise indicated.

Abbreviations: STGs, Standard Treatment Guidelines; UTI, urinary tract infection; WHO, World Health Organization.

a Pediatric UTI therapy recommendations are available in the Tanzania STGs, fourth edition (2013), but not in the fifth edition (2017).

## DISCUSSION

In this large descriptive study of 2 inpatient febrile illness cohorts spanning from 2011 through 2019, we found that broad-spectrum antibacterial use was common both prior to and after admission. Antibacterial selection did not align with blood culture data among a substantial proportion of participants with BSI. Guideline-consistent therapy for pneumonia, UTI, and sepsis increased over time. Our findings highlight mismatches between clinical and laboratory diagnoses and antibacterial management that have implications for patient outcomes, judicious use of antibacterial medications, and guideline-recommended therapy. Additionally, our findings of increasing antimicrobial resistance in Enterobacterales (Supplementary Table 4) suggest that currently available empiric treatment guidelines for BSI may not fully reflect the current epidemiology and antibacterial resistance.

More than 40% of participants self-reported using antibacterials prior to admission for febrile illness. Adults commonly reported use of amoxicillin, amoxicillin-clavulanate, and metronidazole prior to admission (Table 2). Antibacterials are widely available in many areas of Tanzania, often prescribed in the outpatient setting, and commonly obtained with and without prescriptions from pharmacies despite government regulations [3, 29, 30].

The majority of febrile patients enrolled in the 2 study cohorts received inpatient antibacterials, which is appropriate for febrile inpatients. Use of broad-spectrum antibacterials, specifically ceftriaxone, increased between the 2 cohorts. Ceftriaxone was prescribed to >40% of all adult participants, and this is similar to findings in other regions of Tanzania [13, 15]. Ceftriaxone is recommend by guidelines for empiric therapy of pneumonia, sepsis, and UTI. Increased use aligns with increased guideline-consistent therapy for the aforementioned diagnoses [24, 25, 28]. Several participants in cohort 2 had BSIs caused by organisms with intrinsic or acquired resistance to ceftriaxone (Supplementary Tables 3 and 4), and resistance to third- and fourth-generation cephalosporins has been described in Tanzania [5]. Use of ampicillin and gentamicin was common in children and infants. While these antibacterials are guideline consistent, the prevalence of resistance to ampicillin among Enterobacterales in these surveillance cohorts was 81.2% (Supplementary Table 4) and elsewhere in Tanzania is as high as 92.0% [31–33]. These data should inform future revisions to the Tanzanian STGs.

Metronidazole use was common among adults and children and increased between cohort 1 and cohort 2, similar to previous studies (Table 3) [3, 13]. However, generally metronidazole is not indicated as empiric therapy or definitive treatment in community-acquired pneumonia or bacteremia. Use of ampicillin also increased between cohort 1 and cohort 2. This may reflect variation in demographic composition between the 2 study cohorts; cohort 1 included pediatric patients only at MRRH and cohort 2 included pediatric patients at both MRRH and KCMC. Finally, prescription of doxycycline was low despite Q fever and rickettsial diseases being common in northern Tanzania [34, 35].

Among those with BSI, receipt of ineffective therapy due to lack of adequate activity against the microbiologically identified organism, receipt of no inpatient antibacterial therapy or inappropriate enteral therapy, or growth of a resistant organism with demonstrated resistance against the antibacterial prescribed was common. Phenotypic antimicrobial resistance occurred in approximately 10% of all patients with BSI. These inadequate or inappropriate prescribing patterns are likely multifactorial and may be due to outdated treatment guidelines, failure to modify therapy considering culture data, and distrust in microbiological culture results. [11]. Some participants did not receive antibacterial therapy for BSI and this may have occurred due to discharge prior to availability of BSI results, ineffective use of microbiologic results, or clinician choice. Further understanding of physician prescribing practices, educational initiatives, and interventions to refine therapy when culture data are available are needed to better tailor treatment in patients with laboratory-confirmed infections.

Consistency with WHO and Tanzania STGs substantially increased between cohort 1 and cohort 2. This may be due to increased awareness of the guideline recommendations, more accurate clinical diagnoses, or changes in preferred empiric therapy. Adherence to the Tanzanian STGs for pneumonia and UTI was similar to prior studies conducted in the Dodoma and Mbeya regions [13, 14]. Less than a third of participants with clinical diagnosis of UTI received country-specific guideline-consistent therapy; this may be because Tanzanian STGs advise ciprofloxacin or amoxicillin for cystitis, whereas WHO guidance provides additional choices (Supplementary Table 2) [28].

Our study had several limitations. Data collected before admission relied on self-report, are subject to recall bias, and are a poor surrogate of antimicrobial use [21, 36]. Data collection practices slightly differed between the 2 cohorts; specifically, HIV status was self-reported in cohort 1 and case report forms in the 2 cohorts included different pick lists of antimicrobials. The clinical diagnoses given by hospital clinicians were not reevaluated by the study team. Many participants had multiple diagnoses, suggesting diagnostic uncertainty, and antibacterials noted in the record may not have been assigned to a specific diagnosis. Finally, practices at the referral hospitals included in this study may not be generalizable to other hospitals locally or nationally, based upon resources and level of specialty training.

## CONCLUSIONS

We demonstrate that receipt of antibacterials prior to admission and inpatient antibacterial use were common within 2 large cohorts of participants admitted with febrile illness in northern Tanzania. Use of broad-spectrum antibacterials was also common. A substantial proportion of participants with BSIs were treated with ineffective antibacterials. Consistency of antibacterial prescribing with WHO and Tanzanian guidelines improved over time. A global point prevalence survey would provide deeper insight into antimicrobial indications and utilization at these hospitals. Improved diagnostics for febrile illness, data on local antimicrobial resistance patterns, institution-specific clinical guidelines, and provider education may improve rational prescribing practices.

## Supplementary Material

ofad448_Supplementary_Data

## Supplementary Data

Supplementary materials are available at *Open Forum Infectious Diseases* online. Consisting of data provided by the authors to benefit the reader, the posted materials are not copyedited and are the sole responsibility of the authors, so questions or comments should be addressed to the corresponding author.

## Notes

***Author contributions.*** D. B. M., K. G. K., B. F. L., R. M., B. T. M., J. S. N., W. S., J. P. B., M. C., J. T. H., M. J. M., V. P. M., J. A. C., and M. P. R. contributed significantly to the experimental design or its implementation. G. S. M., D. B. M., J. R. E., L. G. T., J. A. C., and M. P. R. contributed to analysis and interpretation of the data. All authors were involved in the writing or editing of the manuscript at any stage and have read and approved the final version.

***Acknowledgments.*** The authors thank persons involved in recruitment, laboratory work, data management, and study administration, including Godfrey S. Mushi, Flora W. Mboya, Lilian E. Ngowi, Winfrida H. Shirima, Michael E. Butoyi, Anna H. Mwalla, Miriam L. Barabara, Ephrasia Mariki, Edna Ngowi, Olterere Salimu, Rither Mhela, Jamal Bashiri, Christopher Swai, Jerome Mlangi, Tumsifu G. Tarimo, Yusuf S. Msuya, Leila J. Sawe, Aaron E. Tesha, Luig J. Mbuya, Edward M. Singo, Stephen Sikumbili, Erica Chuwa, Daniel Mauya, Isaac A. Afwamba, Thomas M. Walongo, Remigi P. Swai, Augustine M. Musyoka, Rose Oisso, Gershom Mmbwambo, Philoteus A. Sakasaka, O. Michael Omondi, Enoch J. Kessy, Alphonse S. Mushi, Robert S. Chuwa, Charles Muiruri, Cynthia A. Asiyo, Frank M. Kimaro, and Francis P. Karia. We also thank the study participants as well as the clinical staff and administration at Kilimanjaro Christian Medical Centre and Mawenzi Regional Referral Hospital for their support during this study.

***Data availability.*** The datasets used or analyzed during the current study are available from the corresponding author on reasonable request.

***Financial support*.** This work was supported by the joint National Institutes of Health (NIH)–National Science Foundation Ecology of Infectious Disease program (grant number R01TW009237); the Research Councils UK, UK Department for International Development, and Biotechnology and Biological Sciences Research Council (grant numbers BB/J010367/1, BB/L018926, BB/L017679, and BB/L018845); the NIH (award number U01 AI062563 and grant number R01 AI121378); and the Bill & Melinda Gates Foundation–funded Typhoid Fever Surveillance in Sub-Saharan Africa Program (grant number OPPGH5231). G. S. M. received support from the Eunice Kennedy Shriver National Institute of Child Health and Human Development (training grant number 1T32HD094671) and the Fogarty International Center of the NIH (grant number D43 TW010543). M. P. R. and D. B. M. received support from the National Institute of Allergy and Infectious Diseases (award number K23 AI116869 and grant number R01 AI155733-S01, respectively).
