# Supplementary material for: Antibacterial Utilization for Febrile Illnesses and Laboratory-Confirmed Bloodstream Infections in Northern Tanzania
Source: Open Forum Infect Dis. 2023 Aug 21;10(8):ofad448. doi: 10.1093/ofid/ofad448 (PMC10468737; doi:10.1093/ofid/ofad448)
Supplement: ofad448_Supplementary_Data [file ofad448_supplementary_data.docx]

**SUPPLEMENTARY DATA**

**Supplementary Table 1.** Antibacterial use collected in standardized questionnaires administered to participants enrolled in two fever surveillance studies, Cohort 1 (2011–2014) and Cohort 2 (2016–2019), northern Tanzania

|  | **Cohort 1 (2011 – 2014)** | **Cohort 2 (2016 – 2019)** |
| --- | --- | --- |
| **Antibacterials reported as used prior to admission** | Amoxicillin-Clavulanate, Ampicillin, Ampicillin-Cloxacillin, Azithromycin, Ceftriaxone, Chloramphenicol, Ciprofloxacin, Cloxacillin, Doxycycline/Tetracycline, Erythromycin, Gentamicin, Metronidazole, Other^*^, Penicillin, Trimethoprim-Sulfamethoxazole, Unknown | Amoxicillin-Clavulanate, Amoxicillin, Azithromycin, Ceftriaxone, Ciprofloxacin, Doxycycline, Erythromycin, Flucloxacillin, Metronidazole, Other (specify), Penicillin, Trimethoprim-Sulfamethoxazole  Antibacterials specified in category of other included: Ampicillin, Ampicillin-Cloxacillin, Cephalexin, Chloramphenicol, Clarithromycin, Gentamicin |
| **Antibacterials prescribed during admission** | Amoxicillin-Clavulanate, Ampicillin, Ampicillin-Cloxacillin, Azithromycin, Ceftriaxone, Cephalexin, Chloramphenicol, Ciprofloxacin, Cloxacillin, Doxycycline/Tetracycline, Erythromycin, Gentamicin, Metronidazole, Other*, Penicillin, Trimethoprim-Sulfamethoxazole, Unknown | Amoxicillin, Ampicillin, Azithromycin, Ceftriaxone, Cephalexin, Chloramphenicol, Ciprofloxacin, Cloxacillin, Doxycycline, Erythromycin, Gentamicin, Metronidazole, Other (specify), Penicillin, Trimethoprim-Sulfamethoxazole  Antibacterials specified in category of other included: Amoxicillin-Clavulanate, Ampicillin-Cloxacillin, Cefepime, Cefotaxime Flucloxacillin, Clarithromycin, Nitrofurantoin, Streptomycin |
| ^*^ The study questionnaire used during Cohort 1 did not include a free text field to further specify which other antibacterials were prescribed. | | |

**Supplementary Table 2.** Antibacterials recommendations from the World Health Organization and the Tanzania Standard Treatment Guidelines (STG) for children and adults with pneumonia, urinary tract infection, or sepsis/bloodstream infection

|  | **Adult Guidelines** | | **Pediatric Guidelines** | |
| --- | --- | --- | --- | --- |
| **Syndrome** | **World Health Organization** | **Tanzania STG** | **World Health Organization** | **Tanzania STG** |
| **Pneumonia** | Ceftriaxone  Amoxicillin  Cotrimoxazole  Doxycycline  Erythromycin  Ampicillin AND gentamicin AND either a macrolide or a respiratory fluoroquinolone | First-line  Amoxicillin  Erythromycin  Ceftriaxone  Second-line  Trimethoprim-sulfamethoxazole  Cloxacillin  Clindamycin  Chloramphenicol  Gentamicin | Amoxicillin  Ampicillin  Cloxacillin  Cloxacillin AND gentamicin | Amoxicillin  Penicillin B  Ampicillin AND gentamicin  Ceftriaxone  If >5 years old: consider erythromycin or azithromycin for *M. pneumoniae* |
| **Urinary Tract Infection** | Trimethoprim-sulfamethoxazole  Amoxicillin-clavulanic acid  Nitrofurantoin  Ciprofloxacin  Ceftriaxone | Ciprofloxacin  Amoxicillin-clavulanic acid | Trimethoprim-sulfamethoxazole  Ampicillin  Amoxicillin  Cephalexin  Ampicillin AND gentamicin  Ceftriaxone | Amoxicillin-clavulanate^*^ |
| **Sepsis or Bloodstream Infection** | Ceftriaxone | Ceftriaxone | Ceftriaxone  Ampicillin AND gentamicin | Ceftriaxone  Ampicillin AND gentamicin |
| Guidelines referenced: World Health Organization IMAI District Clinician Manual – Hospital Care Adolescents and Adults: Guidelines For The Management Of Illnesses With Limited-Resources (Volumes I and II 2012), World Health Organization Updates on the Management of Severe Acute Malnutrition in Infants and Children (2013), World Health Organization Pocket Book of Hospital Care for Children (2013), Tanzania Standard Treatment Guidelines, 4^th^ edition (2014) Tanzania Standard Treatment Guidelines, 5^th^ edition (2017)  ^*^ Pediatric urinary tract infection therapy recommendations are available in the Tanzania STG, 4th edition (2013) but not in the 5^th^ edition (2017) | | | | |

**Supplementary Table 3:** Adjudication of ineffective antibacterial therapy based on organism isolated and prescribed antibacterials among participants enrolled in two fever surveillance studies, Cohort 1 (2011–2014) and Cohort 2 (2016–2019), northern Tanzania

| **Cohort** | **Organism Isolated** | **Inpatient Antibacterials** | **Reason for  Ineffective Therapy^*^** | | | |
| --- | --- | --- | --- | --- | --- | --- |
|  |  |  | **1** | **2** | **3** | **4** |
| 1 | *Salmonella enterica* Typhi | Ampicillin, gentamicin, trimethoprim-sulfamethoxazole | x |  |  | x |
|  | *Salmonella* spp. | Gentamicin, other antibacterial | x |  |  |  |
|  | *Streptococcus pneumoniae* | Gentamicin, trimethoprim-sulfamethoxazole | x |  | x |  |
|  | *Salmonella enterica* Enteritidis | Penicillin | x |  |  |  |
|  | *Escherichia coli* | None |  | x |  |  |
|  | *Escherichia coli* | None |  | x |  |  |
|  | Gram negative rod, non-lactose fermenter | None |  |  | x |  |
|  | *Escherichia coli* | None |  | x |  |  |
|  | *Escherichia coli* | None |  | x |  |  |
|  | *Staphylococcus aureus* | Amoxicillin-clavulanate, trimethoprim-sulfamethoxazole |  |  | x |  |
|  | *Escherichia coli* | Ampicillin, other antibacterial |  |  |  | x |
|  | *Escherichia coli* | Ampicillin, metronidazole |  |  |  | x |
|  | *Escherichia coli* | Ciprofloxacin, metronidazole |  |  |  | x |
| 2 | *Salmonella enterica* Typhi | Erythromycin, metronidazole | x |  |  |  |
|  | *Enterococcus* spp. | Ceftriaxone | x |  |  |  |
|  | *Brucella* spp*.* | Ceftriaxone, metronidazole | x |  |  |  |
|  | *Salmonella enterica* Typhi | Ampicillin, gentamicin, penicillin | x |  |  | x |
|  | *Escherichia coli Morganella morganii* | None |  | x |  |  |
|  | *Streptococcus pneumoniae* | None |  | x |  |  |
|  | *Staphylococcus aureus* | None |  | x |  |  |
|  | *Salmonella enterica* Typhi | None |  | x |  |  |
|  | *Enterobacter aerogenes* | None |  | x |  |  |
|  | *Salmonella enterica* Enteritidis | None |  | x |  |  |
|  | *Escherichia coli* | None |  | x |  |  |
|  | *Staphylococcus aureus* | None |  | x |  |  |
|  | *Klebsiella* spp.,  *Staphylococcus aureus* | Ceftriaxone, metronidazole |  |  |  | x |
|  | *Escherichia coli* | Ceftriaxone, gentamicin |  |  |  | x |
|  | *Escherichia coli* | Ampicillin-cloxacillin, gentamicin |  |  |  | x |
|  | *Escherichia coli* | Azithromycin, ceftriaxone, metronidazole |  |  |  | x |
| ^*^ Ineffective therapy was defined and coded as one or more the following:   1. Participant was prescribed an antibacterial without adequate activity against the microbiologically identified organism (e.g., ceftriaxone for *Pseudomonas* spp.) or intrinsic resistance (e.g., vancomycin for a Gram-negative bacteria or ceftriaxone for *Enterococcus* spp.) 2. No antimicrobial was prescribed to the participant 3. Inappropriate oral therapy prescribed in a participant with an identified bloodstream infection 4. The microbiologically identified organism that generally has susceptibility to the prescribed antibacterial, but the specific isolate demonstrated resistance against the antibacterial the participant was prescribed (e.g., receiving ceftriaxone and *E. coli* isolate is resistant to 3^rd^ generation cephalosporins) | | | | | | |

**Supplementary Table 4:** Antibacterial susceptibility of selected organisms isolated among participants enrolled in two fever surveillance studies, Cohort 1 (2011–2014) and Cohort 2 (2016–2019), northern Tanzania

| **Organism** | **Cohort  (*n* isolates)** | **Susceptibility (% (isolates susceptible/total isolates with complete data))** | | | | | | | | | | | | | | |
| --- | --- | --- | --- | --- | --- | --- | --- | --- | --- | --- | --- | --- | --- | --- | --- | --- |
|  |  | **AMC** | **AMP** | **CFZ** | **FOX** | **CAZ** | **AXO** | **CHL** | **CIP** | **ERY** | **GEN** | **NAL** | **PEN** | **PIP** | **SXT** | **VAN** |
| **Gram-negative Organisms** | | | | | | | | | | | | | | | | |
| *Enterobacterales* | Cohort 1 (32) | 87.5 (28/32) | 18.8 (6/32) | 84.3 (27/32) | 0.0 (0/3) | 100.0 (28/28) | 96.9 (31/32) | 68.8 (22/32) | 96.8 (30/31) | - | 90.3 (28/31) | 80.6 (25/31) | 100.0 (6/6) | 100.0 (6/6) | 18.8 (6/32) | - |
|  | Cohort 2 (20) | 58.6 (10/19) | 15.8 (3/19) | 41.7 (5/12) | 0.0 (0/2) | 86.7 (13/15) | 83.3 (15/18) | 63.2 (12/19) | 75.0 (15/20) | - | 66.7 (10/15) | 75.0 (15/20) | 100.0 (1/1) | 100.0 (1/1) | 20 (4/20) | - |
|  | Overall (52) | 74.5 (38/51) | 17.6 (9/51) | 72.7 (32/44) | 0.0 (0/5) | 95.3 (41/43) | 92.0 (46/50) | 66.7 (34/51) | 88.2 (45/51) | - | 82.6 (38/46) | 78.4 (40/51) | 100.0 (7/7) | 100.0 (7/7) | 19.2 (10/52) | - |
| *Escherichia coli* | Cohort 1 (17) | 76.5 (13/17) | 17.6 (3/17) | 70.6 (12/17) | - | 100.0 (14/14^*^) | 94.0 (16/17) | 64.7 (11/17) | 93.8 (15/16) | - | 81.3 (13/16)^*^ | 75.0 (12/16) | - | - | 5.8 (1/17) | - |
|  | Cohort 2 (10) | 40.0 (4/10) | 0.0 (0/10) | 25.0 (2/8)^*^ | - | 85.7 (6/7)^*^ | 75.0 (6/8)^*^ | 77.8 (7/9) | 60.0 (6/10) | - | 60.0 (6/10) | 50.0 (5/10) | - | - | 0 (0/10) | - |
|  | Overall (27) | 63.0 (17/27) | 11.1 (3/27) | 40.0 (10/25) | - | 95.2 (20/21) | 88.0 (22/25) | 69.2 (18/26)^*^ | 80.8 (21/26) | - | 73.1 (19/26) | 65.4 (17/26) | - | - | 37.0 (1/27) | - |
| *Enterobacter aerogenes* | Cohort 2 (1) | 0.0 (0/1) | 0.0  (0/1) | 100.0 (1/1) | - | - | 100.0 (1/1) | 100.0 (1/1) | 100.0 (1/1) | - | 100.0 (1/1) | 100.0 (1/1) | - | - | 100.0 (1/1) | - |
| *Hafnia alvei* | Cohort 2 (1) | 0.0 (0/1) | 0.0  (0/1) | 100.0 (1/1) | - | - | 100.0 (1/1) | 0.0 (0/1) | 100.0 (1/1) | - | 100.0 (1/1) | 100.0 (1/1) | - | - | 0.0 (0/1) | - |
| *Klebsiella* sp.^1^ | Cohort 1 (3) | 100.0 (3/3) | 0.0  (0/3) | 100.0 (3/3) | 0.0 (0/3) | 100.0 (3/3) | 100.0 (3/3) | 100.0 (3/3) | 100.0 (3/3) | - | 100.0 (3/3) | 100.0 (3/3) | - | - | 66.7 (2/3) | - |
|  | Cohort 2 (2) | 50.0 (1/2) | 0.0  (0/2) | 50.0 (1/2) | 0.0 (0/2) | 50.0 (1/2) | 50.0 (1/2) | 50.0 (1/2) | 100.0 (2/2) | - | 50.0 (1/2) | 100.0 (2/2) | - | - | 0.0 (0/2) | - |
|  | Overall (5) | 80.0 (4/5) | 0.0  (0/5) | 80.0 (4/5) | 0.0 (0/5) | 80.0 (4/5) | 80.0 (4/5) | 80.0 (4/5) | 100.0 (5/5) |  | 80.0 (4/5) | 100.0 (5/5) | - | - | 40.0 (2/5) | - |
| *Salmonella enterica* Typhi^2^ | Cohort 1 (9) | 100.0 (9/9) | 0.0  (0/9) | 100.0 (9/9) | - | 100 (8/8)^*^ | 100.0 (9/9) | 55.6 (5/9) | 100.0 (9/9) | - | 100.0 (9/9) | 77.8 (7/9) | - | - | 0.0 (0/9) | - |
|  | Cohort 2 (3) | 100.0 (3/3) | 33.3 (1/3) | - | - | 100.0 (3/3) | 100.0 (3/3) | 0.0 (0/3) | 66.7 (2/3) | - | - | 100.0 (3/3) | - | - | 0.0 (0/3) | - |
|  | Overall (12) | 100.0 (12/12) | 8.3 (1/12) | 100.0 (9/9)^*^ | - | 100.0 (11/11) | 100.0 (12/12) | 41.7 (5/12) | 91.7 (11/12) | - | 100.0 (9/9)^*^ | 83.3 (10/12) | - | - | 0.0 (0/12) | - |
| *Salmonella* spp. non-Typhi^3^ | Cohort 1 (3) | 100.0 (3/3) | 100.0 (3/3) | 100.0 (3/3) | - | 100.0 (3/3) | 100.0 (3/3) | 100.0 (3/3) | 100.0 (3/3) | - | 100.0 (3/3) | 100.0 (3/3) | 100.0 (2/2)^*^ | 100.0 (2/2)^*^ | 100.0 (3/3) | - |
|  | Cohort 2 (3) | 100.0 (2/2)^*^ | 100.0 (2/2)^*^ | - | - | 100.0 (3/3) | 100.0 (3/3) | 100.0 (3/3) | 100.0 (3/3) | - | 100.0 (1/1)^*^ | 100.0 (3/3) | 100.0 (1/1)^*^ | 100.0 (1/1)^*^ | 100.0 (3/3) | - |
|  | Overall (6) | 100.0 (5/5)^*^ | 100.0 (5/5)^*^ | 100.0 (3/3) | - | 100.0 (6/6) | 100.0 (6/6) | 100.0 (6/6) | 100.0 (6/6) | - | 100.0 (4/4)^*^ | 100.0 (6/6) | 100.0 (3/3)^*^ | 100.0 (3/3)^*^ | 100.0 (6/6) | - |
| **Gram-positive Organisms** | | | | | | | | | | | | | | | | |
| *Streptococcus pneumoniae* | Cohort 1 (3) | - | - | - | - | - | - | 100.0 (3/3) | - | 100.0 (3/3) | - | - | - | - | 0.0 (0/3) | - |
|  | Cohort 2 (2) | - | - | - | - | - | - | 100.0 (2/2) | - | 100.0 (2/2) | - | - | - | - | 0.0 (0/2) | - |
|  | Overall (5) | - | - | - | - | - | - | 100.0 (5/5) | - | 100.0 (5/5) | - | - | - | - | 0.0 (0/5) | - |
| *Staphylococcus aureus* | Cohort 1 (1) | - | - | - | 100.0 (1/1) | - | - | 100.0 (1/1) | - | 100.0 (1/1) | - | - | 0.0 (0/1) | - | 0.0 (0/1) | 100.0 (1/1) |
|  | Cohort 2 (6) | - | - | - | 100.0 (5/5)^*^ | - | - | 100$ (6/6) | - | 33.3 (2/6) | - | - | 16.6 (1/6) | - | 83.3 (5/6) | 80.0 (4/5)^*^ |
|  | Overall (7) | - | - | - | 100.0 (6/6) | - | - | 100.0 (7/7) | - | 42.9 (3/7) | - | - | 14.3 (1/7) | - | 71.4 (5/7) | 83.3 (5/6) |
| *Enterococcus faecalis* | Cohort 2 (1) | - | 100.0 (1/1) | - | - | - | - | - | - | - | - | - | - | - | - | - |
| ^*^ Indicates missing data and/or untested isolates  ^1^ *Klebsiella* species includes *Klebsiella* sp. (n =1) and *Klebsiella pneumoniae* (n = 4)  ^2^ Cefazolin and gentamicin not tested for *S. typhi* in Cohort 2  ^3^ *Salmonella* spp. non-Typhi includes 3 isolates identified as *S. enterica* Enteritidis and 3 identified as *Salmonella* spp.  Abbreviations: number (*n*), amoxicillin-clavulanate (AMC), ampicillin (AMP), cefazolin (CFZ), cefoxitin (FOX), ceftazidime (CAZ), ceftriaxone (AXO), chloramphenicol (CHL), ciprofloxacin (CIP), erythromycin (ERY), gentamicin (GEN), nalidixic acid (NAL), penicillin (PEN), piperacillin (PIP), trimethoprim-sulfamethoxazole (SXT), vancomycin (VAN) | | | | | | | | | | | | | | | | |
